# Prevalence of monoclonal gammopathy of undetermined significance in Eswatini: a population-based study in Africa

**DOI:** 10.1093/jncics/pkae056

**Published:** 2024-07-11

**Authors:** Kara I Cicero, Xolisile Dlamini, Yvonne Mavengere, Jessica Justman, Harriet Nuwagaba-Biribonwoha, Sindisiwe Dlamini, Mxolisi Dlamini, Simphiwe Ngwenyama, Cebisile Ngcamphalala, Andrea Low, Neena M Philip, Wafaa M El-Sadr, Ruben Sahabo, Tesfay Abreha, Sintayehu Temesgen, Nokuthula Mahlalela, Codruta Chiuzan, Yuxuan Chen, Samuel S Pan, Suzanne Lentzsch, Alfred I Neugut

**Affiliations:** Division of Hematology & Oncology, Department of Medicine, University of Washington, Seattle, WA, USA; Clinical Research Division, Fred Hutchinson Cancer Center, Seattle, WA, USA; National Cancer Prevention & Control, Eswatini Ministry of Health, Mbabane, Eswatini; ICAP at Columbia Mailman School of Public Health, Mbabane, Eswatini; Department of Medicine, Vagelos College of Physicians & Surgeons, Columbia University, New York, NY, USA; Department of Epidemiology, Mailman School of Public Health, Columbia University, New York, NY, USA; ICAP at Columbia Mailman School of Public Health, New York, NY, USA; ICAP at Columbia Mailman School of Public Health, Mbabane, Eswatini; Department of Epidemiology, Mailman School of Public Health, Columbia University, New York, NY, USA; Health Laboratory Services, Eswatini Ministry of Health, Mbabane, Eswatini; ICAP at Columbia Mailman School of Public Health, Mbabane, Eswatini; ICAP at Columbia Mailman School of Public Health, Mbabane, Eswatini; ICAP at Columbia Mailman School of Public Health, Mbabane, Eswatini; Department of Epidemiology, Mailman School of Public Health, Columbia University, New York, NY, USA; ICAP at Columbia Mailman School of Public Health, New York, NY, USA; ICAP at Columbia Mailman School of Public Health, New York, NY, USA; Department of Medicine, Vagelos College of Physicians & Surgeons, Columbia University, New York, NY, USA; Department of Epidemiology, Mailman School of Public Health, Columbia University, New York, NY, USA; ICAP at Columbia Mailman School of Public Health, New York, NY, USA; Herbert Irving Comprehensive Cancer Center, Vagelos College of Physicians & Surgeons, Columbia University, New York, NY, USA; ICAP at Columbia Mailman School of Public Health, Mbabane, Eswatini; ICAP at Columbia Mailman School of Public Health, Mbabane, Eswatini; Eswatini Ministry of Health, Mbabane, Eswatini; Eswatini Ministry of Health, Mbabane, Eswatini; Feinstein Institutes for Medical Research, Northwell Health, New York, NY, USA; Herbert Irving Comprehensive Cancer Center, Vagelos College of Physicians & Surgeons, Columbia University, New York, NY, USA; Herbert Irving Comprehensive Cancer Center, Vagelos College of Physicians & Surgeons, Columbia University, New York, NY, USA; Department of Medicine, Vagelos College of Physicians & Surgeons, Columbia University, New York, NY, USA; Herbert Irving Comprehensive Cancer Center, Vagelos College of Physicians & Surgeons, Columbia University, New York, NY, USA; Department of Medicine, Vagelos College of Physicians & Surgeons, Columbia University, New York, NY, USA; Department of Epidemiology, Mailman School of Public Health, Columbia University, New York, NY, USA; Herbert Irving Comprehensive Cancer Center, Vagelos College of Physicians & Surgeons, Columbia University, New York, NY, USA

## Abstract

**Background:**

Although monoclonal gammopathy of undetermined significance (MGUS) and multiple myeloma disproportionately affect Black individuals, few epidemiological studies have been conducted on these plasma cell disorders in Africa. Here we describe the prevalence of MGUS in Eswatini and compare our results to the landmark Olmsted County, Minnesota study.

**Methods:**

Between 2016 and 2017, 13 339 residents of Eswatini participated in the Swaziland HIV Incidence Measurement Survey, from which a nationally representative biorepository was created. Plasma samples were then randomly selected and analyzed for MGUS. MGUS prevalence in Eswatini was compared with that of Olmsted County. In addition, demographic and HIV-related associations with MGUS were assessed.

**Results:**

Of the 515 samples randomly selected, the median age was 50 years (range = 35-80 years); 60% were female; and 38.6% were HIV positive, of whom 82.4% were on antiretroviral therapy. We found that 68 participants had evidence of MGUS, for a prevalence of 13.2%. HIV status was not significantly associated with MGUS (odds ratio = 1.05, 95% confidence interval = 0.62 to 1.77), but among HIV-positive individuals, MGUS was less frequent for patients on antiretroviral therapy (adjusted odds ratio = 0.31, 95% confidence interval = 0.11 to 0.82). The prevalence of conventional MGUS was similar between Eswatini and Olmsted County (3.4% vs 3.2%-3.4%), whereas the incidence of light-chain MGUS was significantly greater in Eswatini (12.3% vs 0.8%).

**Conclusion:**

Our study suggests that the incidence of MGUS is similar between ethnicities and raises the question of whether the current definition of light-chain MGUS reliably reflects a true monoclonal protein precursor state. Perhaps the current definition of light-chain MGUS may be capturing alternate etiologies, such as untreated HIV infection.

Multiple myeloma and its precursor, monoclonal gammopathy of undetermined significance (MGUS), have been well studied in White cohorts but disproportionately affect Black populations (1-5). The cumulative risk of progression is similar between ethnicities (1), which suggests that the ethnic disparities affecting multiple myeloma appear to originate from the initial development of MGUS, rather than from disparate patterns of progression from MGUS to malignancy.

The landmark epidemiological study that described the natural history of MGUS was conducted in Olmsted County, Minnesota (United States), among a 97.3% White population (6). This study found the age-standardized prevalence of MGUS (including light chain) to be 4.2% (7), but subsequent analyses comparing MGUS prevalence in White and Black populations within the United States revealed a 1.3-4 times greater prevalence of MGUS in Black cohorts (1,3,4).

Only two studies have evaluated the prevalence of MGUS in a Black African population. Among Black Ghanaian men, the prevalence of conventional MGUS (defined in the sections that follow) was 5.84% (95% confidence interval [CI] = 4.27 to 7.40) (2) compared with a 2.97% (95% CI = 2.59 to 3.34) prevalence among male individuals of the same age group in the original Olmsted County study (2). In addition, we previously reported the prevalence of MGUS (including light chain) to be 8.03% (95% CI = 5.32 to 10.74) among Black South African men, which was nearly 1.6-fold higher than the prevalence reported in the Olmsted County male population (8).

Our exploratory South African study revealed a possible association between MGUS and HIV status (univariate odds ratio [OR] = 2.39, 95% CI = 0.95 to 5.49; multivariate OR = 2.17, 95% CI = 0.66 to 6.59) (8), but data on the association between MGUS and HIV remain scarce. Only one retrospective study in the United States directly compared the rates of MGUS between HIV-positive and HIV-negative cohorts, finding that 19.3% of the HIV-positive cohort had MGUS compared to 8.2% among the HIV-negative cohort (*P* < .001); notably, however, 13.4% of the HIV-positive patients were tested for MGUS compared with only 3.8% of the HIV-negative patients (9).

To date, no study has examined the prevalence of MGUS in a Black African population inclusive of both sexes. Moreover, no study has prospectively analyzed the prevalence of MGUS within an HIV-positive cohort compared with an HIV-negative cohort.

Eswatini is a small country in southern Africa (Figure 1), with a population of 1.1 million, 97% of whom identify as Black (10), and the world’s highest prevalence of HIV (10). In this study, we determined the prevalence of MGUS in Eswatini and compared our results with the landmark Olmsted County study. In addition, we examined the association between MGUS and HIV status.

**Figure 1.**
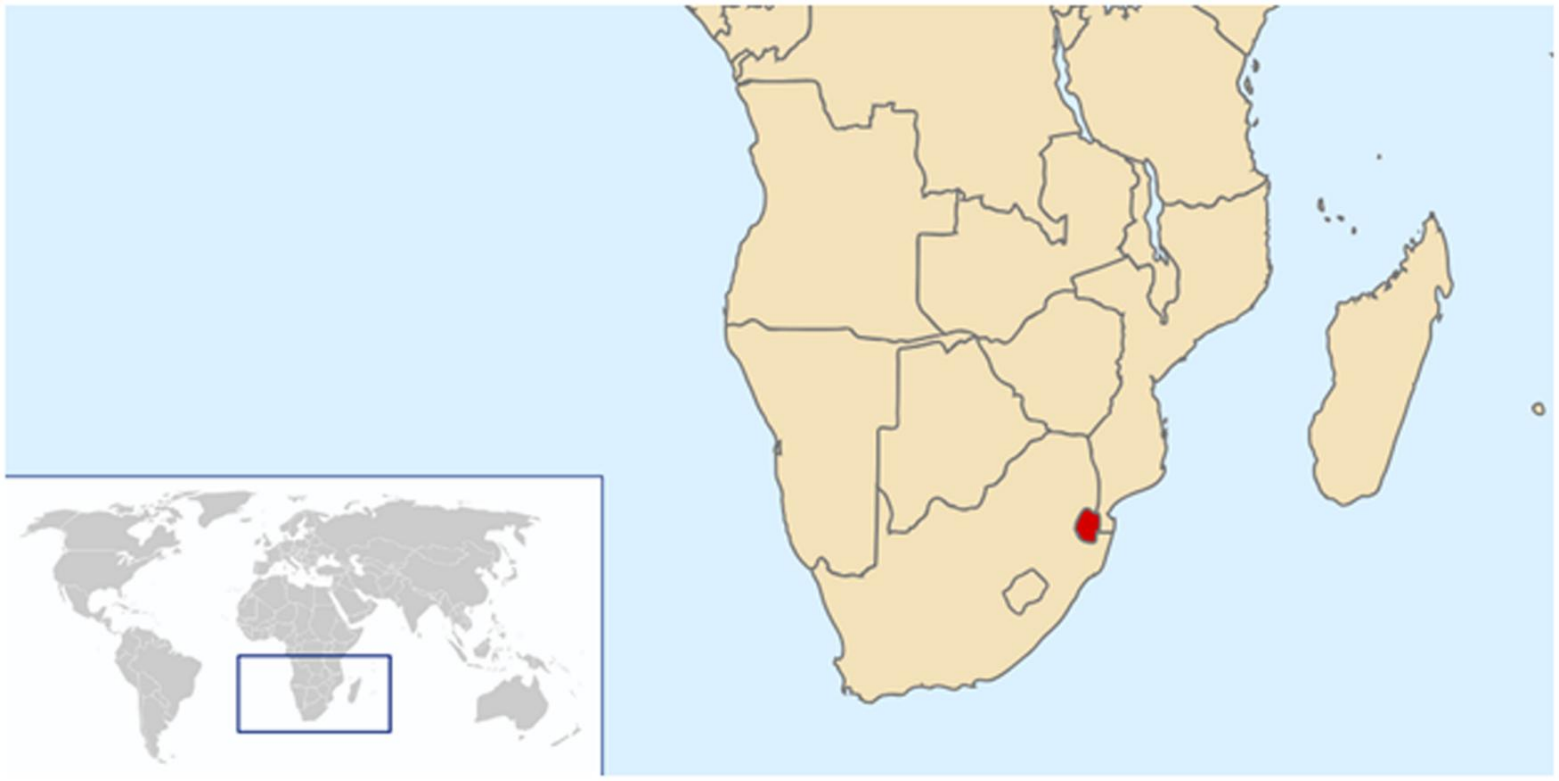
A map of southern Africa, with Eswatini highlighted.

## Methods

The 2016-2017 Swaziland HIV Incidence Measurement Survey (SHIMS2) was a cross-sectional national survey to measure the impact of Eswatini’s national HIV response. SHIMS2 was part of the Population-Based HIV Impact Assessment project, in which HIV-focused surveys and blood specimen collection facilitated assessment of the status of the HIV epidemic in 14 of the most affected countries worldwide (11).

As previously described (12), between August 30, 2016, and March 31, 2017, residents from 5185 randomly selected households were invited to enroll in SHIMS2. Individuals who provided informed consent completed interviews and provided blood samples (N = 15 453). The field teams then transported the blood samples to the national reference laboratory (National Health and Laboratory Services, Mbabane, Eswatini) for processing. All participants were tested for HIV (screening: Determine HIV-1/2, Abbott Molecular, Chicago, IL; confirmatory: Uni-Gold HIV-1/2, Trinity Biotech, County Wicklow, Ireland; tiebreaker: Clearview Complete HIV-1/2, Chembio, Medford, NY ; second confirmatory: Geenius HIV 1/2 Supplemental Assay, Bio-Rad, Hercules, CA). For individuals who tested positive, CD4+ cell counts (Pima CD4 Analyzer, Abbott Molecular, Chicago, IL) and quantitative viral loads (Roche cobas AmpliPrep/cobas TaqMan HIV-1 Test, Roche Molecular Diagnostics, Indianapolis, IN) were measured, and dried blood samples underwent separate qualitative assays for detection of common first-line and second-line antiretroviral agents [high-resolution liquid chromatography coupled with tandem mass spectrometry at the University of Cape Town, South Africa, per Koal et al. (13) methodology]. Plasma aliquots were subsequently frozen at ‒80 °C within 24 hours of collection. All personal health information was removed from samples upon storage, and all survey data were deidentified.

The primary objective of our study was to determine the prevalence of MGUS in Eswatini in comparison to the prevalence reported in Olmsted County. As a secondary objective, we sought to determine the association between MGUS and HIV status. Samples from all SHIMS2 participants older than 35 years of age with known HIV status were included. As we intended a population-based analysis, the only exclusion criterion was participants with less than 1 mL stored plasma, the minimum required for complete testing for MGUS. From this sampling frame, we randomly selected 515 plasma samples. The sample size was calculated to ensure power greater than 80% for an MGUS prevalence estimate assumed to be at least 8% [approximately double the 4.2% rate reported in Olmsted County (7)] and produce a 2-sided 95% confidence interval with a width equal to 0.05 for an estimated prevalence between 8% and 10%. From a sample size of 515, we anticipated 42 MGUS cases and 473 controls based on prevalence estimates from our study in South Africa (8), and we expected 190 participants within our HIV-positive cohort for analysis based on an HIV rate of 37% among individuals older than 35 years of age per SHIMS2 (12). A sample size of 515 would therefore achieve greater than 80% power to detect an odds ratio of 2.4 for the prevalence comparison of HIV-positive vs HIV-negative cohorts, at a *P* value of .05.

Plasma samples were transported while maintaining cold-chain conditions to the Bio-Analytical Research Corporation Laboratory in Johannesburg, South Africa, for protein electrophoresis with reflex immunofixation, as well as free light-chain (FreeLite, Binding Site, UK) and creatinine measurements. MGUS cases were defined using the same criteria as those used in the Olmsted County studies for reliable comparison—that is, 1) monoclonal protein on electrophoresis, regardless of free light chain ratio (conventional MGUS), or 2) abnormal free light chain ratio plus elevation in the appropriate free light chain, despite normal protein electrophoresis (light-chain MGUS) (6,7). Kidney function dictated the delineation for normal values of the free light chain ratio given the kidney’s effect on κ clearance: If the creatinine was within the laboratory’s reference values, a free light chain ratio less than 0.26 or greater than 1.65 was considered abnormal, whereas if the creatinine was greater than the upper limit of the laboratory’s reference values, the renal reference range was used for free light chain ratios (<0.37 or >3.1 was considered abnormal) (14). Following the proposed revision for the definition of light-chain MGUS by the Icelandic group at the American Society of Hematology conference in 2023, a post hoc analysis of the prevalence of MGUS in Eswatini was conducted according to Long et al.’s (15) algorithm.

Data were summarized by group (MGUS positive vs negative) using descriptive statistics. Among MGUS cases, we explored the distribution of isotypes as well as risk stratification for progression to malignancy (7,16). HIV status was the primary exposure investigated; for individuals who were HIV-positive, we further explored the association of CD4+cell count, viral load suppression, and antiretroviral therapy (ART) use with MGUS. We secondarily examined age, sex, and socioeconomic factors [wealth quintiles, determined per SHIMS2 (12)] with the presence of MGUS or as confounders to the association between MGUS and HIV. Historical proportions and means were compared with *z* tests and *t* tests, respectively. Associations between categorical values were calculated with logistic regression or Fisher exact tests and reported as odds ratios with corresponding 95% confidence intervals. All statistical analyses were performed in R, version 4.2.2, statistical software (R Foundation for Statistical Computing, Vienna, Austria) (17) using a 2-sided type I error of .05.

This study was approved by the Institutional Review Board at Columbia University, the Eswatini Health and Human Research Review Board, and Eswatini’s Ministry of Health.

## Results

A total of 13 339 adults provided informed consent for their blood specimens and associated data to be stored for future research. Of these individuals, 4710 were older than 35 years of age and eligible for the current study; 515 individuals were randomly selected from among this group. The demographic characteristics of the study cohort (n = 515) can be found in Table 1. The median age was 50 years (range = 35-80 years), and 60% of the participants were female (n = 309); 199 (38.6%) were HIV positive, of whom 82.4% (n = 164) were on ART, and 8.5% (n = 17) had a CD4+ cell count below 200/mL. These findings are consistent with overall national estimates in Eswatini (12). Twenty-eight participants (5.4%) had renal insufficiency, whereby the renal reference range was used for free light chain ratios.

**Table 1.** Demographic characteristics of adults in Eswatini, 2016-2017, whose plasma samples were analyzed for the presence of monoclonal gammopathy of undetermined significance (n = 515)

Characteristic (n* *=* *515)	No. (%)
Age, y	
35-39	100 (19.4)
40-49	146 (28.3)
50-59	123 (23.9)
60-69	80 (15.5)
70-79	50 (9.7)
80-89	16 (3.1)
Sex	
Male	206 (40)
Female	309 (60)
Wealth quintile	
1 (lowest)	122 (23.7)
2	104 (20.2)
3	139 (27.0)
4	76 (14.8)
5 (highest)	74 (14.4)
HIV status	
Positive	199 (38.6)
Current ART use^a^ (% of total HIV-positive individuals)	164 (82.4)
Viral load suppression, HIV RNA <1000/mL (% of total HIV-positive individuals)	164 (82.4)
CD4 cell count <200/mL (% of total HIV-positive individuals)	17 (8.5)
CD4 cell count 200-500/mL (% of total HIV-positive individuals)	63 (31.7)
CD4 cell count >500/mL (% of total HIV-positive individuals)	119 (60.0)
Negative	316 (61.4)

a Current ART use based on ART detection assays. ART = antiretroviral therapy.

### Prevalence

The diagnostic criteria for MGUS were met in 13.2% (95% CI = 10.5% to 16.5%) of the participants (n = 68). κ was involved in 93% (n = 63) of cases, whereas λ was involved in 7% (n = 5). Most (84% [n = 57]) of the MGUS cases were the light-chain isotype. The median (interquartile range [IQR]) free light chain ratio for individuals with light-chain MGUS involving κ (n = 55) was 1.83 (1.74-1.97) and involving λ (n = 2) was 0.17 (0.09-0.23). Of the 11 (16%) individuals with conventional MGUS, 8 were IgG and 3 were IgA; 4 were considered very low risk for progression to malignancy, 6 were low risk, 1 was intermediate risk, and none were considered high risk.

Table 2 shows the MGUS prevalence distribution directly compared between the Eswatini and Olmsted County cohorts, with prevalence stratified by age, sex, and isotype (light-chain vs conventional). The Olmsted County studies included only individuals older than 50 years of age (6,7). The rate of MGUS in Eswatini for individuals older than 50 years of age was 15.6% (95% CI = 11.3% to 20.0%), which was 3.7 times greater than the prevalence reported in Olmsted County (*P* < .001). Light-chain MGUS was found in 12.3% (n = 33) of those over 50 years of age in Eswatini compared with 0.8% in Olmsted County (7). Of individuals older than 50 years of age, 3.4% (n = 9) had conventional MGUS, a rate similar to that reported for conventional MGUS in Olmsted County (3.2%-3.4%) (6,7). By using the revised Icelandic definition in the post hoc analysis, MGUS prevalence in Eswatini decreased by 76% since light-chain MGUS decreased from 57 cases to 5 cases. Only 3.11% (n = 16) of the Eswatini cohort now met criteria for MGUS, of which light-chain MGUS comprised 31% of total MGUS cases.

**Table 2.** MGUS prevalence in Eswatini and Olmsted County, Minnesota, stratified by age, sex, and light-chain vs conventional isotype^a^

Panel A. Eswatini, 2016-2017									
	Light-chain MGUS			Conventional MGUS			Any MGUS		
	Male, n/N (%)	Female, n/N (%)	Total, n/N (%)	Male, n/N (%)	Female, n/N (%)	Total, n/N (%)	Male, n/N (%)	Female, n/N (%)	Total, n/N (%)
50-59 y	4/42 (9.52)	12/81 (14.81)	16/123 (13)	0/42 (0)	1/81 (1.23)	1/123 (0.813)	4/42 (9.52)	13/81 (16.05)	17/123 (13.82)
60-69 y	5/29 (17.24)	5/51 (9.80)	10/80 (12.5)	1/29 (3.45)	2/51 (3.92)	3/80 (3.75)	6/29 (20.69)	7/51 (13.73)	13/80 (16.25)
70-79 y	0/21 (0)	6/29 (20.69)	6/50 (12)	2/21 (9.52)	1/29 (3.45)	3/50 (6)	2/21 (9.52)	7/29 (24.14)	9/50 (18)
80-89 y	0/4 (0)	1/12 (8.33)	1/16 (6.25)	0/4 (0)	2/12 (16.67)	2/16 (12.5)	0/4 (0)	3/12 (25)	3/16 (18.75)
Total	9/96 (9.38)	24/173 (13.87)	33/269 (12.27)	3/96 (3.13)	6/173 (3.47)	9/269 (3.35)	12/96 (12.5)	30/173 (17.34)	42/269 (15.61)
Age-standardized prevalence	22/206 (10.68)	35/309 (11.33)	—	5/206 (2.43)	6/309 (1.94)	—	27/206 (13.11)	41/309 (13.27)	—
Prevalence standardized for age and sex	—	—	57/515 (11.1)	**—**	**—**	11/515 (2.14)	**—**	**—**	68/515 (13.20)

**Panel B. Olmsted County, MN, 1995-2001** (7)									
	Light-chain MGUS			Conventional MGUS			Any MGUS		
	Male, %	Female, %	Total, %	Male, %	Female, %	Total, %	Male, %	Female, %	Total, %
50-59 y	0.60	0.40	0.50	2.20	1.30	1.70	2.80	1.70	2.20
60-69 y	1.00	0.50	0.80	3.80	2.60	3.10	4.80	3.10	3.90
70-79 y	1.70	0.70	1.10	5.50	4.30	4.80	7.20	5.00	5.90
80-89 y	1.00	1.50	1.30	8.90	6.70	7.40	9.90	8.20	8.70
Total	1.00	0.60	0.80	3.80	3.10	3.40	4.80	3.70	4.20
Age-standardized prevalence	1.00	0.60	—	4.10	2.90	—	5.10	3.50	—
Prevalence standardized for age and sex	—	—	0.8	**—**	**—**	3.4	**—**	**—**	4.2

a MGUS = monoclonal gammopathy of undetermined significance.

### Demographic and HIV-related associations

The odds of MGUS increased by 2.4% with each year of age (*P* = .02); this association remained significant when adjusted for sex, socioeconomic status, and HIV status (multivariate OR = 1.03, 95% CI = 1.01 to 1.05). Neither sex nor wealth quintile was found to be significantly associated with MGUS (Table 3).

**Table 3.** Associations of demographic characteristics with MGUS in Eswatini^a^

**All** **participants**	MGUS positive (n* *=* *68)	MGUS negative (n* *=* *447)	Univariable odds ratio (95% confidence interval)	*P*	Multivariable odds ratio (95% confidence interval)	Adjusted *P*
Age, median (IQR), y	56.5 (45.0-63.5)	50 (41-60)	1.024 (1.00 to 1.044)	.02	1.026 (1.01 to 1.05)	.02
Sex				.96	1.05 (0.62 to 1.78)	.85
Male, No. (%)	27 (39.7)	179 (40.0)	0.986 (0.59 to 1.66)	—	—	—
Female, No. (%)	41 (60.3)	268 (60.0)	(Referent)	—	—	—
Wealth quintile, mean (SD)	2.57 (1.26)	2.79 (1.36)	0.88 (0.73 to 1.08)	.22	0.92 (0.75 to 1.78)	.37
HIV status	—	—	—	.85	1.32 (0.75 to 2.32)	.34
Positive, No. (%)	27 (39.7)	172 (38.5)	1.05 (0.62 to 1.77)	—	—	—
Negative, No. (%)	41 (60.3)	275 (61.5)	(Referent)	—	—	—

a IQR = interquartile range; MGUS = monoclonal gammopathy of undetermined significance.

HIV status was not significantly associated with MGUS in univariate or multivariate models (univariate OR = 1.05, 95% CI = 0.62 to 1.77; multivariate OR = 1.32, 95% CI = 0.75 to 2.32) (Table 4). Among HIV-positive participants (Table 3), however, the odds of MGUS were 69% lower among participants on ART (OR = 0.31, 95% CI = 0.11 to 0.82) compared with participants not on ART when adjusted for age, sex, and wealth quintile. Furthermore, MGUS was 2.8 times more frequent among HIV-positive participants not on ART than among participants on ART (95% CI = 1.1 to 7.2, *P* = .03) and 2.3 times more frequent than among HIV-negative participants (95% CI = 1.0 to 5.6, *P* = .056); MGUS among individuals with HIV on ART did not differ from HIV-negative individuals (data not shown). In contrast, viral load suppression and CD4+ cell counts were not associated with MGUS. In addition, 92.6% (n = 25) of individuals with MGUS and HIV infection had a light-chain isotype compared with 78.1% (n = 32) of individuals with MGUS but without HIV infection; 7.4% (n = 2) of participants with MGUS and HIV had a conventional isotype compared with 22.0% (n = 9) of participants with MGUS but without HIV (Supplementary Table 1, available online).

**Table 4.** Associations of HIV status and MGUS in Eswatini

HIV-positive participants	**MGUS positive (n* = *27), No.** **(%)**	MGUS negative (n* = *172), No. (%)	Univariable odds ratio (95% CI)	*P*	**Adjusted** ^a^ **odds ratio (95% CI)**	Adjusted *P*
CD4 > 500/mL	12 (44.4)	107 (62.2)	0.84 (0.17 to 4.13)	.83	0.89 (0.17 to 4.69)	.89
CD4 200-500/mL	13 (48.1)	50 (29.1)	1.95 (0.40 to 9.6)	.41	1.68 (0.32 to 8.82)	.54
CD4 < 200/mL	2 (7.4)	15 (8.7)	(Referent)	—	(Referent)	—
On ART	18 (66.7)	146 (84.9)	0.36 (0.13 to 1.06)	.04	0.31 (0.11 to 0.82)	.02
Not on ART	8 (29.6)	23 (13.4)	(Referent)	—	(Referent)	—
Data missing	1 (3.7)	3 (1.7)	—	—	—	—
Viral load suppressed (<1000/mL)	20 (74.1)	144 (83.7)	0.56 (0.20 to 1.71)	.27	0.46 (0.17 to 1.24)	.13
Viral load not suppressed	7 (25.9)	28 (16.3)	(Referent)	—	(Referent)	—

a Adjusted for age, sex, and wealth quintile. ART = antiretroviral therapy; CI = confidence interval; MGUS = monoclonal gammopathy of undetermined significance.

**Figure 2.**
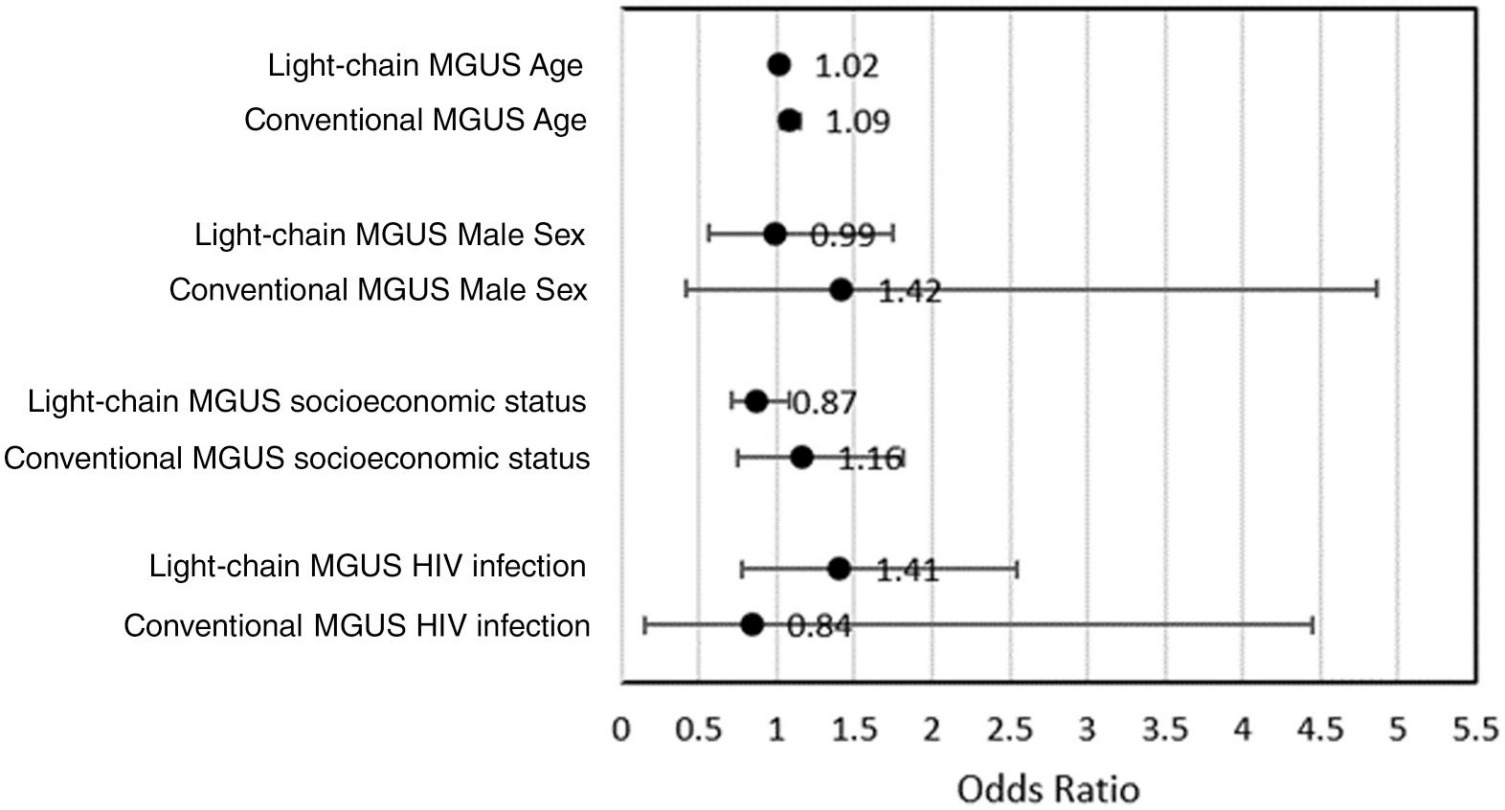
Subgroup analysis for the odds of MGUS in relation to demographic characteristics. MGUS = monoclonal gammopathy of undetermined significance.

Figure 2 demonstrates the isotype subgroup analysis (conventional vs light-chain) for the odds of MGUS in relation to demographic characteristics. Conventional MGUS, but not light-chain MGUS, remained significantly associated with age (conventional MGUS OR = 1.09, 95% CI = 1.03 to 1.15; light-chain MGUS OR = 1.02, 95% CI = 1.00 to 1.04). Male individuals trended toward higher rates of conventional MGUS (OR = 1.42, 95% CI = 0.4 to 4.9), but no difference was apparent with light-chain MGUS (OR = 0.99, 95% CI = 0.56 to 1.75). The higher prevalence of conventional MGUS favored higher wealth quintiles (OR = 1.16, 95% CI = 0.75 to 1.82), while light-chain MGUS did not (OR = 0.87, 95% CI = 0.71 to 1.08). HIV infection trended toward an increased risk of light-chain MGUS (OR = 1.41, 95% CI = 0.77 to 2.55) but a decreased risk of conventional MGUS (OR = 0.84, 95% CI = 0.16 to 4.45).

## Discussion

In this population-based analysis of a nationally representative sample from Eswatini, we initially found the prevalence of total MGUS cases to be more than 3-fold higher than that in Olmsted County. The rates of conventional MGUS, however, were similar between Eswatini and Olmsted County, which differs from previously reported disparities of conventional MGUS between Black and White Americans (3,4), as well as Ghanaian and Olmsted County men (2). The significant differences in prevalence reported here were largely driven by light-chain MGUS, which was more than 13 times higher in Eswatini than in Olmsted County. The high prevalence of HIV in Eswatini in and of itself does not account for the high prevalence of total MGUS cases, as the majority of people living with HIV in Eswatini were on ART, and only individuals living with HIV and not currently on ART had significantly higher odds of having MGUS.

Rather than a biological difference from ethnic ancestry, our findings may instead reflect the idea that the current definition of MGUS, particularly for the light-chain isotype, may not be capturing a true monoclonal precursor state. The definition for light-chain MGUS is based on an “abnormal” free-light-chain ratio. The median free light chain ratio for individuals with κ light-chain MGUS by standard criteria in Eswatini was 1.83 (IQR = 1.74-1.97), which was only minimally above the “normal” laboratory reference of 1.65. The values for an “abnormal” free light chain ratio and assumed clonality are based on Olmsted County data (7), and it is possible that different populations may require different reference values for free light chain ratios to reflect clonality. This idea is further supported by the substantial reduction in light-chain cases when using the Icelandic revised criteria for light-chain MGUS. Free light chain ratios may be confounded by factors unrelated to clonality, such as chronic inflammation.

We found that only people living with HIV and not currently on ART had significant odds of having MGUS. Three explanations are suggested. First, perhaps individuals with treated HIV have functional immune systems resembling their HIV-negative counterparts and thus have adequate tumor surveillance to control aberrant plasma cells. In that case, we would expect viral load suppression and CD4+ cell counts to have an analogous relationship to ART and MGUS; however, similar to the findings reported by Genet et al. (18), we did not find a significant association between CD4+ cell count or viral load suppression and MGUS. It should be noted, though, that the number of HIV-positive individuals included in this study may have limited the ability to show significant associations. Second, it is possible that ART directly affects the plasma cell clone because preclinical models have demonstrated that protease inhibitors have antimyeloma cell line effects (19). Moreover, more than half of participants with MGUS and HIV have previously been shown to have a decrease in monoclonal protein concentration after initiation of ART (20), and HIV-positive patients with MGUS have been previously found to be 2.7 times less likely to progress to hematologic malignancies than their HIV-negative counterparts (9). Finally, chronic inflammation, such as in the setting of HIV infection, may confound the free light chain ratio. In other words, current diagnostic criteria for light-chain MGUS may detect monotypic rather than monoclonal gammopathies related to chronic B-cell activation in the setting of viral infection. We found a trend in the association between HIV infection and light-chain, but not conventional, MGUS supporting this possibility, although subgroups were not adequately powered to detect significance.

The prospect that cases defined by standard criteria here as light-chain MGUS do not, in reality, reflect a clonal precursor state is further suggested by the isotype subgroup analysis. For example, it is well established that both age and male sex are risk factors for MGUS (6,7), and we would therefore expect a higher prevalence associated with these factors if a monoclonal precursor state were captured here. However, we found that only conventional MGUS, but not light-chain MGUS, significantly increased with age; in addition, conventional MGUS, but not light-chain MGUS, trended toward increased prevalence with male sex. It is important to note, however, that such risk factors have been validated only within studies of largely White populations. Caution should be taken in drawing any conclusions from the subgroup analyses reported here, though, because the sample sizes were small and not adequately powered to determine these relationships with confidence.

There were three main limitations in our study. First, we had plasma available for analysis rather than serum. In clinical practice, serum is used to detect monoclonal gammopathy because fibrinogen may appear as a monoclonal protein on plasma electrophoresis. However, fibrinogen has been shown to interfere with paraprotein identification, in only 2.3% of plasma electrophoresis samples (21), and in those cases, subsequent immunofixation differentiated fibrinogen from true heavy-chain monoclonal gammopathy. In our study, fibrinogen was not found to interfere, as every monoclonal protein detected on protein electrophoresis corresponded to an immunoglobulin on immunofixation. Our results should therefore be reliable comparators to prior studies in which serum protein electrophoresis was used. Second, we used a historic rather than contemporaneous cohort for our comparison group. Third, given the deidentified, cross-sectional nature of the available biorepository, repeat laboratory studies for validation, bone marrow biopsy to confirm underlying plasma cell clonality, and longitudinal follow up for progression were not possible.

The findings from our study motivate the need for a larger longitudinal study to further investigate baseline characteristics and the natural history of MGUS in contemporaneous cohorts of different ethnic groups, as well as individuals with and without HIV. The role of ART on plasma cells is particularly interesting and should be further investigated. Moreover, comparing light-chain MGUS cases defined by criteria that use free light chain ratios to more specific analyses for clonality, such as serum matrix-assisted laser desorption/ionization with time-of-flight mass spectrometry or bone marrow pathology, will help elucidate whether current free light chain diagnostic criteria are generalizable; perhaps the revised light-chain diagnostic criteria proposed by Long et al. (15) should be more widely used to avoid overdiagnosis of this entity.

In conclusion, our study ultimately deepens our epidemiological understanding of MGUS and multiple myeloma, diseases for which major ethnic disparities were thought to exist. Moreover, our study raises the important question of whether the current definition of light-chain MGUS reliably reflects a true monoclonal protein precursor state.

## Supplementary Material

pkae056_Supplementary_Data

## Data Availability

The data generated in this study are available upon request from the corresponding author.

## References

[1] Landgren O , Gridley G, Turesson I, et al Risk of monoclonal gammopathy of undetermined significance (MGUS) and subsequent multiple myeloma among African American and white veterans in the United States. Blood. 2006;107(3):904-906. 16210333 10.1182/blood-2005-08-3449 PMC1895893

[2] Landgren O , Katzmann JA, Hsing AW, et al Prevalence of monoclonal gammopathy of undetermined significance among men in Ghana. Mayo Clin Proc. 2007;82(12):1468-1473. 18053453 10.1016/S0025-6196(11)61089-6

[3] Landgren O , Graubard BI, Katzmann JA, et al Racial disparities in the prevalence of monoclonal gammopathies: a population-based study of 12,482 persons from the National Health and Nutritional Examination Survey. Leukemia. 2014;28(7):1537-1542. 24441287 10.1038/leu.2014.34 PMC4090286

[4] Landgren O , Graubard BI, Kumar S, et al Prevalence of myeloma precursor state monoclonal gammopathy of undetermined significance in 12372 individuals 10-49 years old: a population-based study from the National Health and Nutrition Examination Survey. Blood Cancer J. 2017;7(10):e618. 29053158 10.1038/bcj.2017.97 PMC5678222

[5] Surveillance, Epidemiology, and End Results Program. *Cancer Stat Facts: Myeloma*. https://seer.cancer.gov/statfacts/html/mulmy.html. Accessed July 2021.

[6] Kyle RA , Therneau TM, Rajkumar SV, et al Prevalence of monoclonal gammopathy of undetermined significance. N Engl J Med. 2006;354(13):1362-1369. 16571879 10.1056/NEJMoa054494

[7] Dispenzieri A , Katzmann JA, Kyle RA, et al Prevalence and risk of progression of light-chain monoclonal gammopathy of undetermined significance: a retrospective population-based cohort study. Lancet. 2010;375(9727):1721-1728. 20472173 10.1016/S0140-6736(10)60482-5 PMC2904571

[8] Cicero KI , Joffe M, Patel M, et al Prevalence of monoclonal gammopathy of undetermined significance in black South African men. Cancer Epidemiol Biomarkers Prev. 2022;31(12):2192-2198. 36126958 10.1158/1055-9965.EPI-22-0525

[9] Jou E , Gligich O, Chan AC, et al Retrospective study of the prevalence and progression of monoclonal gammopathy in HIV positive versus HIV negative patients. Hematol Oncol. 2017;35(1):64-68. 26205037 10.1002/hon.2247

[10] Eswatini. https://www.cia.gov/the-world-factbook/static/c166966c1b8164ed4649b6f985f3b31a/WZ-summary.pdf. Accessed July 2021.

[11] Justman JE , Mugurungi O, El-Sadr WM. HIV population surveys—bringing precision to the global response. N Engl J Med. 2018;378(20):1859-1861. 29768142 10.1056/NEJMp1801934

[12] Government of the Kingdom of Eswatini. *Swaziland HIV Incidence Measurement Survey 2 (SHIMS2)*. Mbabane: Government of the Kingdom of Eswatini; 2016-2017. https://phia.icap.columbia.edu/wp-content/uploads/2019/05/SHIMS2_Final-Report_05.03.2019_forWEB.pdf. Accessed July 2021.

[13] Koal T , Burhenne H, Römling R, et al Quantification of antiretroviral drugs in dried blood spot samples by means of liquid chromatography/tandem mass spectrometry. Rapid Commun Mass Spectrom. 2005;19(21):2995-3001. 16193530 10.1002/rcm.2158

[14] Hutchison CA , Plant T, Drayson M, et al Serum free light chain measurement aids the diagnosis of myeloma in patients with severe renal failure. BMC Nephrol. 2008;9:11. 18808676 10.1186/1471-2369-9-11 PMC2564915

[15] Long TE , Rögnvaldsson S, Thorsteinsdottir S, et al Revised definition of free light chains in serum and light chain monoclonal gammopathy of undetermined significance: results of the Istopmm Study. Blood. 2023;142(suppl 1):535.

[16] Rajkumar SV , Kyle RA, Therneau TM, et al Serum free light chain ratio is an independent risk factor for progression in monoclonal gammopathy of undetermined significance. Blood. 2005;106(3):812-817. 15855274 10.1182/blood-2005-03-1038 PMC1895159

[17] R Core Team. R: A Language and Environment for Statistical Computing. Vienna, Austria: R Foundation for Statistical Computing; 2022.

[18] Genet P , Sutton L, Chaoui D, et al Prevalence of monoclonal gammopathy in HIV patients in 2014. J Int AIDS Soc. 2014;17(4 suppl 3):19649. 25394153 10.7448/IAS.17.4.19649 PMC4224897

[19] Mendez-Lopez M , Sutter T, Driessen C, et al HIV protease inhibitors for the treatment of multiple myeloma. Clin Adv Hematol Oncol. 2019;17(11):615-623. 31851164

[20] Amara S , Dezube BJ, Cooley TP, et al HIV-associated monoclonal gammopathy: a retrospective analysis of 25 patients. Clin Infect Dis. 2006;43(9):1198-1205. 17029142 10.1086/508351

[21] Davison AS , Darn SM, Sodi R. Can lithium-heparin plasma be used for protein electrophoresis and paraprotein identification? Ann Clin Biochem. 2006;43(Pt 1):31-34. 16390607 10.1258/000456306775141821

