# Supplementary material for: Prevalence of monoclonal gammopathy of undetermined significance in Eswatini: a population-based study in Africa
Source: JNCI Cancer Spectr. 2024 Jul 11;8(4):pkae056. doi: 10.1093/jncics/pkae056 (PMC11310106; doi:10.1093/jncics/pkae056)
Supplement: pkae056_Supplementary_Data [file pkae056_supplementary_data.docx]

Supplementary Table 1. MGUS cases stratified by isotype and HIV infection.

|  | **Light Chain MGUS** | **Non-FLC Chain MGUS** |
| --- | --- | --- |
| **HIV+** | 25 (92.59%) | 2 (7.41%) |
| **HIV-** | 32 (78.05%) | 9 (21.95%) |
